# Study of Vibronic and Cationic Features of p-Diethoxybenzene via REMPI, Hole-Burning, and MATI Spectroscopy

**DOI:** 10.3390/ijms27083362

**Published:** 2026-04-09

**Authors:** Xiateng Qin, Yan Zhao, Zhonghua Ji, Changyong Li, Suotang Jia

**Affiliations:** 1 State Key Laboratory of Quantum Optics Technologies and Devices, Institute of Laser Spectroscopy, Shanxi University, Taiyuan 030006, China; 18734558738@163.com (X.Q.); jzh@sxu.edu.cn (Z.J.); tjia@sxu.edu.cn (S.J.); 2 Department of Physics and Electronics Engineering, Jinzhong University, Jinzhong 030619, China; zhaoy@jzxy.edu.cn; 3 Collaborative Innovation Center of Extreme Optics, Shanxi University, Taiyuan 030006, China

**Keywords:** p-diethoxybenzene, rotamer, REMPI spectroscopy, hole-burning, MATI spectroscopy, adiabatic ionization energy, Franck–Condon simulation

## Abstract

Phenetole derivatives with dual ethoxy substituents exhibit rich conformational diversity and complex vibronic characteristics, making them important model compounds for understanding substituent effects on molecular structure and spectroscopy. In this work, we systematically investigated the stable rotamers, vibronic spectra, and cationic ground-state features of p-diethoxybenzene (PDEB) using resonance-enhanced multiphoton ionization (REMPI), UV-UV hole-burning (HB), and mass-analyzed threshold ionization (MATI) spectroscopies, combined with density functional theory (DFT) calculations. The ground-state potential energy surface (PES) of PDEB was calculated at the B3LYP/6-311++G(d,p) level, identifying eight rotamers with distinct statistical weights and relative energies. Hole-burning spectroscopy resolved two dominant rotamers (cis/up–up and trans/up–down) in the supersonic molecular beam, with their S_1_←S_0_ transition origins determined as 33,824 cm^−1^ and 33,613 cm^−1^, respectively. Franck-Condon simulations of the vibronic transitions showed excellent agreement with the experimental REMPI spectra, enabling precise assignment of substituent and benzene ring vibrational modes. MATI experiments yielded accurate adiabatic ionization energies (AIEs) of the cis and trans rotamers as 59,629 ± 5 cm^−1^ and 59,432 ± 5 cm^−1^, respectively, and identified active cationic vibrational modes in the D_0_ state. Geometric parameters of PDEB in the S_0_, S_1_, and D_0_ states were calculated at the B3PW91/aug-cc-pVTZ, TD-B3PW91/aug-cc-pVTZ, and UB3PW91/aug-cc-pVTZ levels, revealing structural evolution during electronic excitation and ionization. The effects of ethoxy substituent orientation on molecular energy, vibrational frequencies, and ionization energy are discussed, and differences in spectral characteristics between PDEB and its meta isomer (MDEB) are compared. This work provides a comprehensive spectral and structural database for p-diethoxybenzene and deepens the understanding of structure–property relationships in diethoxybenzene isomers.

## 1. Introduction

Phenetole (ethoxybenzene) and its derivatives are widely used in pharmaceutical chemistry, materials science, liquid crystal synthesis, electrochemistry, and fine chemical manufacturing. The presence of flexible ethoxy groups (−OC_2_H_5_) in these molecules leads to multiple rotamers due to internal rotation around the C(benzene)-O and O-C(ethyl) bonds, and this conformational diversity directly affects their physical, chemical, and spectral properties [1,2,3]. Understanding the relationship between molecular conformation and spectroscopic behavior is fundamental to both basic chemical physics and practical applications in molecular design.

As a symmetric disubstituted phenetole derivative, diethoxybenzene has three positional isomers (ortho, meta, para) with different substituent spatial orientations, resulting in significant differences in rotamer distribution, vibronic transitions, and ionization characteristics [3]. Among these, p-diethoxybenzene (PDEB) possesses a symmetric molecular skeleton with two ethoxy groups at the para positions of the benzene ring, exhibiting higher structural symmetry and more regular conformational and spectral features. This makes PDEB an ideal model molecule for studying the effects of dual ethoxy substituents on molecular spectroscopy and structure.

Resonance-enhanced multiphoton ionization (REMPI) spectroscopy is a powerful technique for investigating the vibronic structure of excited states in gas-phase molecules, particularly for identifying low-concentration rotamers in supersonic molecular beams [4,5,6,7,8,9]. UV-UV hole-burning (HB) spectroscopy [10,11,12,13,14,15,16], as an important complementary technique to REMPI, can effectively resolve overlapping vibronic spectra of different rotamers by selectively depleting the ground-state population of a specific rotamer, and is considered the gold standard for rotamer discrimination in molecular spectroscopy. Mass-analyzed threshold ionization (MATI) [17,18,19,20,21,22,23,24,25,26,27] or Zero electron Kinetic energy (ZEKE) [28,29,30,31,32,33] spectroscopy, with its high energy resolution, enables accurate measurement of adiabatic ionization energies (AIEs) and vibrational features of the cationic ground state (D_0_), and is widely applied in studies of molecular ionization dynamics and cationic structure. The combination of these three spectroscopic techniques with quantum chemical calculations has become the mainstream approach for revealing conformational diversity and electronic structure in flexible substituted benzene derivatives [2,3,10,34,35,36,37].

In recent years, the spectral and structural characteristics of phenetole derivatives have been extensively studied. Bernstein and coworkers first reported the REMPI spectrum of phenetole and identified its stable rotamers using supersonic jet spectroscopy [38]. Ferres et al. investigated the molecular structure of phenetole using microwave spectroscopy and DFT calculations, predicting two rotamers (trans and gauche) [39]. Grotemeyer and coworkers systematically characterized the vibronic structure of the S_1_ excited state and D_0_ cationic state of trans phenetole via REMPI and MATI spectroscopy [40]. For diethoxybenzene isomers, our group recently reported the first comprehensive study on the rotamers, vibronic spectra, and cationic features of m-diethoxybenzene (MDEB), identifying two dominant rotamers (down–up and down–down) and determining their adiabatic ionization energies (AIEs) as 62,419 ± 5 cm^−1^ and 63,378 ± 5 cm^−1^, respectively [3]. However, research on p-diethoxybenzene (PDEB) remains limited. To date, no systematic investigation into its rotamer distribution or the vibrational features of its S_1_ and D_0_ states has been reported. Due to its symmetric para-substitution, PDEB exhibits a more regular conformational landscape, making it an ideal model for understanding how substituent position influences the structural and spectroscopic properties of diethoxybenzene isomers.

In this work, we generated a supersonic molecular beam of PDEB and recorded its one-color REMPI spectrum along with rotamer-resolved hole-burning spectra. Ground-state rotamers were identified through DFT calculations, and Franck–Condon simulations of the S_1_←S_0_ transition enabled assignment of the experimental vibronic bands. Two-color MATI spectroscopy was employed to determine the adiabatic ionization energies of the dominant cis and trans rotamers and to obtain vibrational spectra of their D_0_ cationic states. Geometric parameters in the S_0_, S_1_, and D_0_ states were calculated to reveal structural changes upon electronic excitation and ionization. The effects of ethoxy orientation on molecular energy, vibrational frequencies, and ionization energy are discussed, and a comparison between PDEB and MDEB highlights the role of substitution pattern. This work provides a comprehensive understanding of structure–property relationships in diethoxybenzene isomers and lays a foundation for future studies on related molecular systems.

## 2. Results and Discussion

### 2.1. Theoretical Rotamers of PDEB in the Ground State (S_0_)

**Figure 1:**
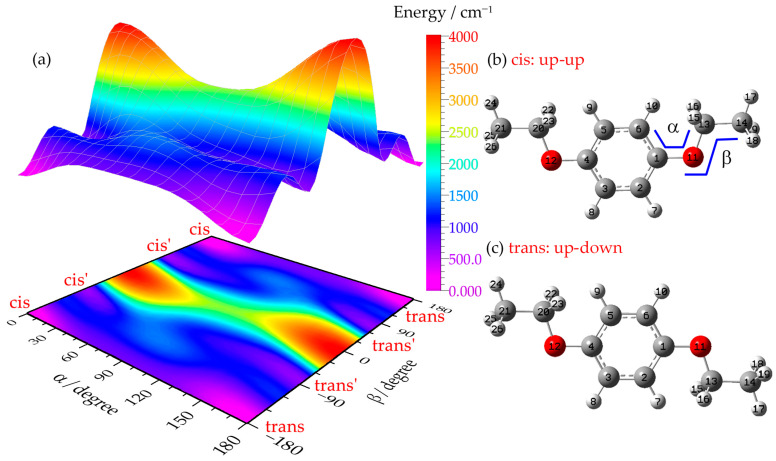
PES calculated at the B3LYP/6-311++G(d,p) level for the S_0_ state of PDEB (**a**) and its stable conformations (**b**,**c**) with atom numbering. The dihedral angles of the ethoxy groups (C6-C1-O11-C13 and C1-C11-O13-C14) determine the rotamer configuration.

p-Diethoxybenzene features two identical ethoxy groups at the para positions of the benzene ring. Each ethoxy group can undergo internal rotation around the C(1)-O(11) and O(11)-C(13) bonds (Figure 1), leading to the formation of multiple possible rotamers [2,3,38,39,40,41]. To explore the conformational landscape of PDEB, we calculated the ground-state (S_0_) potential energy surface (PES) at the B3LYP/6-311++G(d,p) level, systematically scanning the dihedral angles of the ethoxy groups.

**Figure 2:**
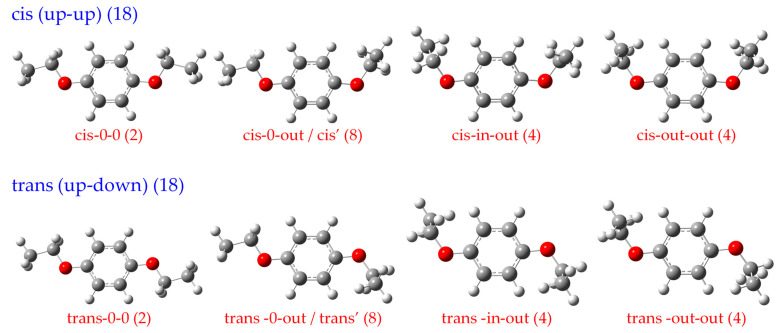
All 8 possible rotamers of PDEB in the S_0_ state and their statistical weights (in parentheses). The rotamers are classified into cis (up–up) and trans (up–down) types based on the relative orientation of the two ethoxy groups. The ethyl moiety orientations are labeled as 0 (in the benzene ring plane), in (toward the ring plane), and out (away from the ring plane).

Figure 1 shows the calculated potential energy surface obtained by fixing one ethoxy group in the ring plane and scanning the two dihedral angles formed by the other ethoxy group relative to the benzene ring. Local energy minima are clearly identified when both ethoxy groups lie in the ring plane (cis and trans isomers), as well as when the ethyl group is tilted out of the plane by approximately ±90° (cis’ and trans’ isomers). To locate all minimum-energy structures theoretically, we fixed one ethoxy group with its ethyl moiety out of the ring plane and scanned the two dihedral angles formed by the other ethoxy group relative to the ring plane, yielding another potential energy surface (see Figure S1 in the Supplementary Materials). The results confirm the existence of eight possible conformers for para-diethoxybenzene, which were classified into cis (up–up) and trans (up–down) types according to the relative orientation of the two ethoxy groups with respect to the ring plane (Figure 2). The cis rotamers include four conformations (0-0, 0-out, in–out, out–out) with both ethoxy groups on the same side of the benzene ring plane, while the trans rotamers also include four conformations (0-0, 0-out, in–out, out–out) with the two ethoxy groups on opposite sides. The nomenclature is based on the orientation of the ethyl moiety: “0” indicates the ethyl group lies in the benzene ring plane, “in” indicates orientation toward the ring plane, and “out” indicates orientation away from the ring plane.

The statistical weights of the eight rotamers were calculated according to their conformational degeneracy, the sum of which is 36 (Figure 2). The cis (0-0) rotamer possesses the lowest relative energy and represents the most stable conformation of PDEB. To accurately compare the relative populations of each rotamer under supersonic molecular beam conditions, we calculated the relative zero-point-level energies of the ground-state rotamers using two different DFT functionals and basis sets (Table 1). The calculation results demonstrate consistent energy ordering across different theoretical levels: the cis (0-0) rotamer has the lowest energy (set as 0 cm^−1^), followed by the trans (0-0) rotamer (54–66 cm^−1^), while rotamers with ethyl moieties oriented out of the benzene ring plane exhibit significantly higher energies (500–1300 cm^−1^).

According to the Maxwell–Boltzmann distribution, only the two lowest-energy rotamers (cis 0-0 and trans 0-0) possess significant populations in the supersonic molecular beam, as the supersonic expansion process effectively cools the molecules and high-energy rotamers are almost entirely depopulated. This prediction is fully consistent with the experimental results obtained from hole-burning spectroscopy (Section 2.2). The energy difference between cis and trans rotamers (~60 cm^−1^) is comparable to that observed in related systems such as p-dimethoxybenzene [42,43,44] and p-ethoxyphenol [41], suggesting similar conformational preferences in para-disubstituted benzene derivatives with alkoxy groups.

### 2.2. Vibronic and Hole-Burning Spectra of PDEB (S_1_←S_0_ Transition)

**Figure 3:**
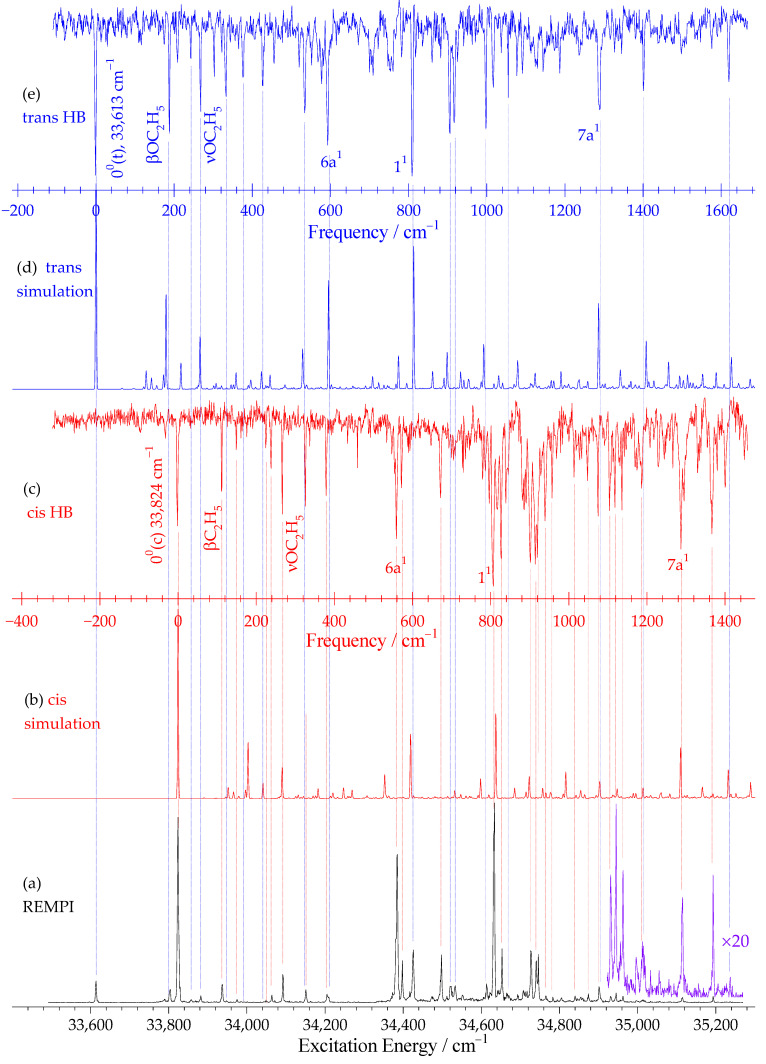
The one-color REMPI spectrum of PDEB (**a**); the Franck–Condon simulated spectra of the S_1_←S_0_ 0_0_^0^ transition for the cis (**b**) and trans (**d**) rotamers; the hole-burning (HB) spectra with the probe laser fixed at 33,824 cm^−1^ (cis, (**c**)) and 33,613 cm^−1^ (trans, (**e**)).

The one-color REMPI spectrum of PDEB in the supersonic molecular beam was measured in the energy range of 33,500–35,300 cm^−1^ (Figure 3a). The spectrum exhibits a series of well-resolved vibronic bands, with two strong peaks at 33,824 cm^−1^ and 33,613 cm^−1^ identified as the 0_0_^0^ transition origins (electronic origins without vibrational excitation) of the two dominant rotamers. Based on the theoretical predictions in Section 2.1, these origins are assigned to the cis (up–up) and trans (up–down) rotamers, respectively. The energy separation of 211 cm^−1^ between these two origins is comparable to that reported for p-ethoxyphenol (97 cm^−1^) [41] and p-dimethoxybenzene (221 cm^−1^) [42,43,44]. Notably, the excitation energy of the cis configuration is consistently higher than that of the trans configuration, highlighting that the relative orientation of alkoxy groups exerts a significant influence on the electronic transition energy.

Due to the overlapping of vibronic bands from different rotamers in the REMPI spectrum, UV-UV hole-burning (HB) spectroscopy was employed to resolve rotamer-specific vibronic spectra. The probe laser was fixed at the 0_0_^0^ origins of the two rotamers (33,824 cm^−1^ for cis, 33,613 cm^−1^ for trans), while the pump laser was scanned to obtain the HB spectra (Figure 3c,e). The HB spectra show characteristic “hole” features at the vibronic bands of the targeted rotamer with minimal cross-interference between the two rotamers, although a few cis and trans bands remain nearly overlapping in the higher vibrational energy region. This confirms that the two 0_0_^0^ origins in the REMPI spectrum belong to two independent rotamers (cis and trans) and validates the theoretical prediction that only these two rotamers have significant populations under supersonic jet conditions.

Franck-Condon simulations of the S_1_←S_0_ vibronic transitions were performed for both rotamers based on the optimized geometries and vibrational frequencies calculated at the TD-B3PW91/aug-cc-pVTZ level (Figure 3b,d). The simulated spectra show good agreement with the experimental REMPI and HB spectra, enabling reliable assignment of the observed vibronic bands. Table 2 lists the observed band positions, shifts from the electronic origins, calculated frequencies, and their assignments. The numbering system for the normal vibrations follows that used by Varsanyi and Szoke [45,46] for benzene derivatives, which is based on Wilson’s notations [47].

The low-frequency region (<300 cm^−1^) is dominated by vibrations involving the ethoxy substituents. The bands at 113 cm^−1^ (cis) and 190 cm^−1^ (trans) are assigned to βC_2_H_5_ (in-plane bending of the ethyl group) and βOC_2_H_5_ (in-plane bending of the OC_2_H_5_ moiety), respectively. These modes are sensitive to the orientation of the ethoxy groups and show distinct frequencies for the two rotamers, consistent with observations in p-ethoxyphenol [41] and m-diethoxybenzene [42,43,44]. The bands around 268–269 cm^−1^ in both rotamers correspond to νOC_2_H_5_ (stretching vibrations involving the C-OC_2_H_5_ linkage).

The medium-frequency region (500–1000 cm^−1^) contains benzene ring deformation modes that are characteristic of para-disubstituted benzenes. The 6b^1^ and 6a^1^ vibrations, which involve in-plane ring deformation, appear at 538 cm^−1^ and 591 cm^−1^ for the trans rotamer, and at 561 cm^−1^ (6a^1^) for the cis rotamer. The breathing mode (1^1^) appears at 810–813 cm^−1^ for both rotamers, with the cis rotamer showing slightly lower frequency (810 cm^−1^) compared to the trans (813 cm^−1^). This mode is particularly important as it involves synchronous expansion and contraction of the benzene ring and is sensitive to the electronic effects of substituents.

The high-frequency region (>900 cm^−1^) contains combination bands and overtones, as well as fundamental modes such as 9a^1^ (1121 cm^−1^ for cis), 7a^1^ (1291–1287 cm^−1^), and various combination bands involving the breathing mode with substituent vibrations (e.g., 1^1^βC_2_H_5_ at 922 cm^−1^ for cis, 1^1^βOC_2_H_5_ at 1000 cm^−1^ for trans). The excellent agreement between experimental and calculated frequencies (typical deviation < 10 cm^−1^) validates the computational methodology and confirms the reliability of the spectral assignments.

### 2.3. MATI Spectra and Cationic Features of PDEB (D_0_←S_1_ Transition)

**Figure 4:**
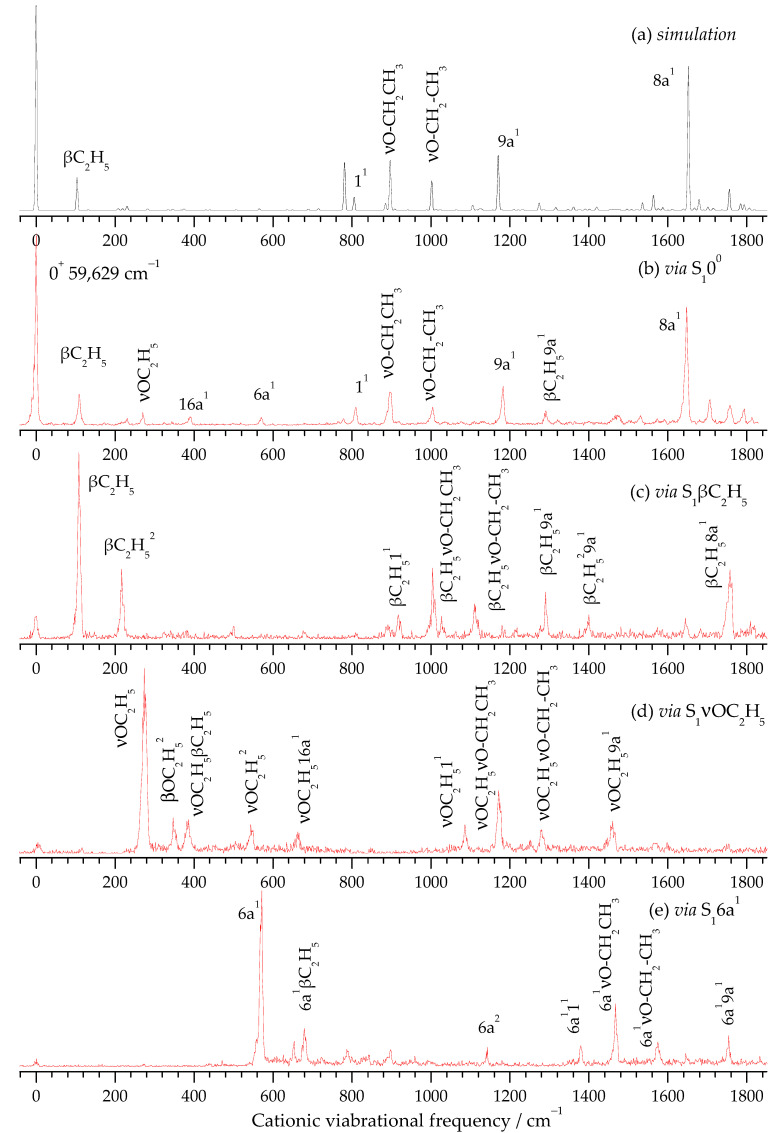
Simulated spectrum of the D_0_ ← S_1_0^0^ transition of the PDEB cis rotamer (**a**) and its MATI spectra via the intermediate states S_1_0^0^ (**b**), S_1_βC_2_H_5_ (**c**), S_1_νOC_2_H_5_ (**d**), and S_1_6a^1^ (**e**).

**Figure 5:**
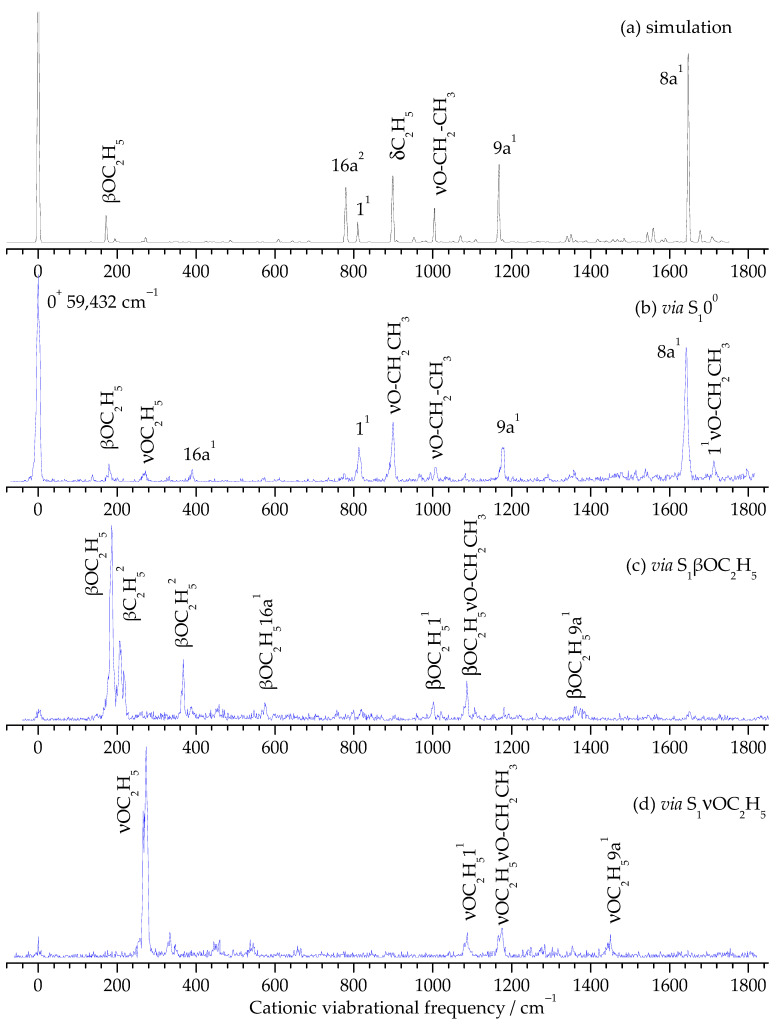
Simulated spectrum of the D_0_ ← S_1_0^0^ transition of the PDEB trans rotamer (**a**) and its MATI spectra via the intermediate states S_1_0^0^ (**b**), S_1_βOC_2_H_5_ (**c**), and S_1_νOC_2_H_5_ (**d**).

The adiabatic ionization energies (AIEs) of the cis and trans rotamers of PDEB were determined by two-color MATI spectroscopy. Figure 4 shows the MATI spectra of the cis rotamer recorded via different intermediate vibrational levels in the S_1_ state, while Figure 5 presents the corresponding spectra for the trans rotamer. The sharp 0^+^ bands (vibrationless cation) appear at 59,629 ± 5 cm^−1^ for the cis rotamer and 59,432 ± 5 cm^−1^ for the trans rotamer, establishing these values as the respective AIEs. The energy difference of 197 cm^−1^ between the two rotamers is comparable to the difference in their S_1_ origins (211 cm^−1^), suggesting that the relative stability ordering is maintained upon ionization.

The MATI spectra exhibit rich vibrational structure corresponding to active modes of the PDEB cation in its ground electronic state (D_0_). Table 3 lists all observed vibrational bands, calculated frequencies, and assignments. The assignments are based on comparison with calculated vibrational frequencies at the UB3PW91/aug-cc-pVTZ level, as well as with previously reported data for structurally related molecules such as p-dimethoxybenzene [42], p-ethoxyphenol [41], and p-methoxyphenol [48].

When the MATI spectra are recorded via different intermediate vibrational levels, a clear propensity for maintaining the vibrational mode of the intermediate level is observed, consistent with the Franck–Condon principle and previous observations in similar systems [49,50]. For example, when the S_1_ 6a^1^ level is used as the intermediate state, the 6a^1^ vibration of the cation appears as the most intense band (Figure 4e for cis, Table 3). This behavior confirms that the molecular geometries and vibrational coordinates of the S_1_ and D_0_ states are similar, as further supported by the geometric parameters discussed in Section 3.2.

The low-frequency region of the MATI spectra (<300 cm^−1^) is dominated by substituent vibrations. For the cis rotamer, bands at 109 cm^−1^ and 270 cm^−1^ are assigned to βC_2_H_5_ and νOC_2_H_5_, respectively. For the trans rotamer, additional bands at 179 cm^−1^ (βOC_2_H_5_) and 206 cm^−1^ (βC_2_H_5_^2^ overtone) appear, reflecting the different symmetry and vibrational coupling in the trans configuration. The observation of overtones and combination bands (e.g., βC_2_H_5_^2^ at 217 cm^−1^ for cis, βOC_2_H_5_^2^ at 367 cm^−1^ for trans) indicates moderate anharmonicity in these low-frequency modes.

The medium-frequency region (300–800 cm^−1^) contains ring deformation modes. The 16a^1^ out-of-plane ring vibration appears at 390 cm^−1^ for both rotamers, showing minimal sensitivity to ethoxy orientation. The in-plane ring deformation 6a^1^ appears at 570 cm^−1^ (cis) and is notably absent in the trans spectra when recorded via the 0^0^ level, showing great sensitivity to ethoxy orientation.

The high-frequency region (>800 cm^−1^) contains the breathing mode (1^1^) at 810–813 cm^−1^, the νO-CH_2_CH_3_ stretching at 896–899 cm^−1^, the 9a^1^ in-plane CH bending at 1183–1177 cm^−1^, and the 8a^1^ ring stretching at 1647–1642 cm^−1^. Numerous combination bands involving these fundamentals with substituent modes are also observed, particularly when higher intermediate levels are used. The excellent agreement between experimental and calculated frequencies (typical deviation < 10 cm^−1^ for fundamentals) validates the computational methodology and confirms the reliability of the assignments.

## 3. Discussion

### 3.1. Comparison of Rotamer Stability in PDEB and MDEB

The conformational preferences of diethoxybenzene isomers provide valuable insights into the effects of substituent position on molecular structure. For PDEB, our calculations predict eight possible rotamers, with the cis (0-0) and trans (0-0) forms being significantly lower in energy than all other conformers (by >400 cm^−1^). This energy gap ensures that only these two rotamers have significant populations under supersonic jet conditions, as confirmed by our HB experiments.

For the meta isomer MDEB, our recent study identified thirteen possible rotamers, with two dominant forms (down–up and down–down) observed experimentally [5]. The larger number of rotamers in MDEB reflects the asymmetric substitution pattern, which reduces symmetry and creates more distinct local minima on the potential energy surface. The energy differences between the two observed MDEB rotamers (~74 cm^−1^) are comparable to those in PDEB (~60 cm^−1^), suggesting similar rotational barriers in both systems.

The statistical weights shown in Figure 2 for PDEB (total 36) reflect the conformational degeneracy arising from the two equivalent ethoxy groups and the symmetry of the para substitution. This is in contrast to MDEB, where the asymmetric substitution leads to different degeneracy factors for different rotamer families. These conformational preferences have important implications for the interpretation of spectroscopic data and for understanding the physical chemistry of these molecules in various environments.

### 3.2. Molecular Geometry in the S_0_, S_1_, and D_0_ States

Table 4 and Table 5 list the optimized geometric parameters of the cis and trans rotamers of PDEB in the S_0_, S_1_, and D_0_ states, calculated at the B3PW91/aug-cc-pVTZ, TD-B3PW91/aug-cc-pVTZ, and UB3PW91/aug-cc-pVTZ levels, respectively. Analysis of these geometric parameters reveals systematic structural changes upon electronic excitation and ionization that are characteristic of benzene derivatives [29,30].

Upon S_1_←S_0_ excitation, the benzene ring undergoes significant expansion. The C-C bond lengths increase by 0.017–0.036 Å for the cis rotamer and 0.014–0.027 Å for the trans rotamer. The largest increases occur at bonds adjacent to the substituent positions (C_2_-C_3_ and C_5_-C_6_), reflecting the quinoidal character of the S_1_ state [31,32]. Concurrently, the C_1_-O_11_ and C_4_-O_12_ bonds shorten by ~0.02 Å, indicating increased double-bond character due to enhanced conjugation between the oxygen lone pairs and the aromatic π-system in the excited state. The C-O-C angles open by ~2.0°, suggesting a rehybridization at the oxygen atoms upon electronic excitation.

Upon D_0_←S_1_ ionization, further structural changes occur. The ring C-C bonds show alternating patterns of lengthening and shortening: bonds involving the substituted carbons (C_1_-C_2_, C_3_-C_4_, C_4_-C_5_, C_6_-C_1_) generally lengthen by 0.008–0.011 Å, while bonds not directly connected to substituents (C_2_-C_3_, C_5_-C_6_) shorten significantly by 0.051–0.058 Å. This pattern reflects the distribution of positive charge in the cation, which is stabilized by the electron-donating ethoxy groups [33]. The C-O bonds shorten further (by ~0.035 Å), indicating even stronger conjugation in the cationic state. The C-O-C angles continue to open, reaching ~122° in the D_0_ state.

The point group symmetries of the two rotamers vary during electronic excitation and ionization. The cis rotamer maintains approximately C2v symmetry in the S_0_ and D_0_ states but reduces to Cs in the S_1_ state due to slight asymmetries in the excited-state geometry. The trans rotamer has C2h symmetry in the S_0_ and D_0_ states but becomes C_1_ in the S_1_ state. These symmetry reductions reflect the different ways in which the two ethoxy groups respond to the changed electronic structure upon excitation and ionization.

### 3.3. Effect of Substituent Orientation on Vibrational Frequencies

Comparison of the vibrational frequencies for cis and trans rotamers reveals the influence of ethoxy group orientation on molecular vibrations. In the S_1_ state (Table 2), most ring modes show only small differences between rotamers: the 1^1^ mode differs by 3 cm^−1^ (810 vs. 813 cm^−1^), and the 7a^1^ mode differs by 4 cm^−1^ (1291 vs. 1287 cm^−1^). These small differences indicate that the intrinsic ring vibrations are relatively insensitive to the relative orientation of the substituents, consistent with observations in other disubstituted benzenes.

In contrast, substituent-sensitive modes show larger variations. The βOC_2_H_5_ mode appears at 190 cm^−1^ in the trans rotamer but is not observed in the cis rotamer, where it may be obscured or have different activity. The νOC_2_H_5_ mode shows nearly identical frequencies (268–269 cm^−1^) for both rotamers, suggesting that this stretching vibration is primarily determined by local bonding rather than global conformation.

In the D_0_ state (Table 3), similar trends are observed. Ring modes such as 16a^1^ (390 cm^−1^ for both), 1^1^ (810–813 cm^−1^), and 9a^1^ (1183–1177 cm^−1^) show minimal rotamer dependence. Substituent modes show somewhat larger variations: βC_2_H_5_ (109 cm^−1^) appears solely in the cis MATI spectrum, whereas βOC_2_H_5_ (179 cm^−1^) is only observed in the trans MATI spectrum. These characteristic differences serve as reliable diagnostic markers to identify the conformational distribution in future spectroscopic studies.

### 3.4. Transition Energies and Ionization Energies

The measured transition energies (S_1_←S_0_) and ionization energies (IE) of *cis*- and *trans*-p-diethoxybenzene, obtained in this work, reveal significant substituent effects relative to benzene (Table 6). The S_1_←S_0_ transition energies for *cis*- and *trans*-p-diethoxybenzene are 33,824 cm^−1^ and 33,613 cm^−1^, respectively, corresponding to red shifts (ΔE_1_) of −4262 cm^−1^ and −4473 cm^−1^ compared to benzene (38,086 cm^−1^). These substantial red shifts indicate that the ethoxy substituents stabilize the excited S_1_ state more effectively than the methoxy groups in p-dimethoxybenzene (ΔE_1_ = −4234 cm^−1^ for cis and −4455 cm^−1^ for trans), consistent with the stronger electron-donating character of the ethoxy group.

In terms of ionization energies, *cis*- and *trans*-p-diethoxybenzene exhibit IEs of 59,629 cm^−1^ and 59,432 cm^−1^, respectively, which are 14,928 cm^−1^ and 15,125 cm^−1^ lower than that of benzene (74,557 cm^−1^). Notably, the IE of *trans*-p-diethoxybenzene is slightly lower than that of the *cis* isomer, suggesting that the *trans* conformer has a more electron-rich aromatic ring and thus a lower ionization energy. This trend is also observed in the p-dimethoxybenzene isomers, where the *trans* conformer has a lower IE (60,563 cm^−1^) than the *cis* form (60,772 cm^−1^).

Compared to the meta-diethoxybenzene isomers (down-up: IE = 62,419 cm^−1^; down-down: IE = 63,378 cm^−1^), the para-substituted isomers show significantly lower ionization energies. This can be attributed to the stronger electron-donating effect of the ethoxy groups in the para position, which more effectively stabilizes the cationic state formed upon ionization. The combined analysis of transition and ionization energies confirms that the ethoxy substituents in p-diethoxybenzene exert a strong electron-donating influence, modulating both the excited-state and cationic state energies in a conformation-dependent manner.

## 4. Experimental and Theoretical Methods

### 4.1. Experimental Methods

The solid sample of *p*-diethoxybenzene (purity 97%, Shanghai Aladdin Biochemical Technology Co., Ltd., Shanghai, China) was used without further purification. The sample was heated to approximately 140 °C to generate sufficient vapor pressure. The vapor was seeded in krypton carrier gas at 3 bar and expanded into the source chamber through a pulsed valve (Parker Series 9, 0.5 mm orifice, Parker Hannifin Corporation, Cleveland, OH, USA) operated with a pulse width of 160 μs and a repeat frequency of 10 Hz. The pressures of the source and ionization chambers were maintained at approximately 10^−4^ Pa and 10^−6^ Pa, respectively. The resulting supersonic molecular beam was collimated by a 1 mm skimmer prior to entering the ionization chamber, where it interacted with laser radiation. Ions traveled through a 48 cm field-free region and were detected by a pair of stacked microchannel plates.

For resonance-enhanced multiphoton ionization (REMPI) experiments, a dye laser (CBR-D-24, Sirah Lasertechnik GmbH, Gottingen, Lower Saxony, Germany; Pyrromethene 597, Exciton Inc., Dayton, OH, USA. All dyes used in this study are sourced from this company) was employed, which was pumped by the second harmonic (532 nm) of a Q-smart 850 Nd:YAG laser (Quantel, Les Ulis, Essonne, France). The output of the dye laser was further frequency-doubled using β-barium borate (BBO) crystals. The timing of the pulsed valve, lasers, and data acquisition was precisely controlled by a digital delay generator (DG645, Stanford Research Systems, Inc., Sunnyvale, CA, USA). REMPI spectra were recorded over a range of approximately 1700 cm^−1^ via segmented scanning (5–10 nm per segment). Laser power was maintained at approximately 10 μJ using neutral density filters.

UV-UV hole-burning (HB) spectroscopy was utilized to identify individual rotamers. A strong pump laser system (PrecisionScan-D dye laser, Sirah Lasertechnik GmbH, Gottingen, Lower Saxony, Germany; Pyrromethene 597, ~1.5 mJ), pumped by the second harmonic (532 nm) of a second Q-smart 850 Nd:YAG laser, and a weak probe laser (CBR-D-24, ~10 μJ) interacted with the molecular beam with a 150 ns delay. The probe wavelength was fixed at a selected REMPI peak (e.g., the electronic origin), while the pump wavelength was scanned. Depletion of the ground-state population of a specific rotamer by the pump laser resulted in a reduction in the probe ion signal, yielding rotamer-resolved HB spectra.

Two-color mass-analyzed threshold ionization (MATI) experiments were performed using the aforementioned laser systems with suitable dyes (LDS 751 dissolved in ethanol or dimethyl sulfoxide (DMSO) as the solvent for the PrecisionScan-D dye laser). Molecules were excited to long-lived high-*n* Rydberg states. A delayed pulsed electric field sequence was applied: −0.7 V/cm at 100 ns to deflect prompt ions, and +143 V/cm at 32 μs to induce field ionization via the Stark effect [48]. The resulting threshold ions were mass-analyzed and detected using a multichannel scaler (SR430, Stanford Research Systems, Inc., Sunnyvale, CA, USA). Each data point was averaged over 300 laser shots, and wavelengths were calibrated using a wavemeter (High-Finesse WS-7, HighFinesse GmbH, Offenburg, Germany).

### 4.2. Theoretical Methods

All calculations were performed using the Gaussian 16 program package [54]. Geometry optimizations and vibrational frequency calculations for the S_0_, S_1_, and D_0_ states were carried out at the B3PW91/aug-cc-pVTZ [55,56,57], TD-B3PW91/aug-cc-pVTZ [58,59] and UB3PW91/aug-cc-pVTZ [55,56,57] levels, respectively. The average value <S^2^> in the D_0_ state is 0.75 after spin annihilation (ca. 0.757 before annihilation), confirming that spin contamination is negligible and the use of UDFT is well justified. The ground state potential energy surface was calculated at the B3LYP/6-311++G(d,p) [60] level as a pragmatic choice balancing computational cost and accuracy, and relative energies of the eight identified rotamers were further evaluated using different functionals and basis sets, including B3LYP [60] and B3PW91 [55,56], in combination with 6-311++G(d,p) and aug-cc-pVTZ basis sets [57] to ensure robustness. Adiabatic ionization energies were also predicted using the G4 and CBS-QB3 composite methods prior to experiments to guide laser dye selection. Franck–Condon simulations of the S_1_←S_0_ and D_0_←S_1_ transitions were performed based on the B3PW91/aug-cc-pVTZ-optimized geometries. The scaling factors for S_1_ (0.97) and D_0_ (0.98) were individually optimized by comparing theoretical vibrational frequencies with experimental data, as the systematic errors of the computational method differ between the neutral excited state and the cation ground state. These factors account for anharmonicity and basis set limitations.

## 5. Conclusions

We have conducted a comprehensive investigation of the rotamers, vibronic spectra, and cationic features of p-diethoxybenzene using REMPI, UV-UV hole-burning, and MATI spectroscopies combined with DFT calculations. Eight possible rotamers of PDEB are predicted theoretically, but only the two lowest-energy rotamers (cis/up–up and trans/up–down) have significant populations under supersonic molecular beam conditions. Hole-burning spectroscopy unambiguously confirms the existence of these two rotamers and resolves their individual vibronic spectra. The S_1_←S_0_ transition origins are determined as 33,824 ± 2 cm^−1^ for the cis rotamer and 33,613 ± 2 cm^−1^ for the trans rotamer. Franck–Condon simulations based on TD-DFT calculations show excellent agreement with experimental spectra, enabling detailed assignment of vibronic bands including substituent-sensitive modes (βC_2_H_5_, βOC_2_H_5_, νOC_2_H_5_) and benzene ring modes (6b, 6a, 1, 9a, 7a).

The adiabatic ionization energies are measured as 59,629 ± 5 cm^−1^ for the cis rotamer and 59,432 ± 5 cm^−1^ for the trans rotamer. These values are significantly lower than those of p-dimethoxybenzene and p-ethoxyphenol, demonstrating the enhanced electron-donating ability of the ethoxy group compared to methoxy and hydroxy groups. Furthermore, the AIEs of p-diethoxybenzene are consistently lower than those of m-diethoxybenzene by 3000–4000 cm^−1^, directly confirming that the para-ethoxy group is a stronger electron donor than its meta counterpart—a trend fully consistent with the expected resonance effects.

MATI spectra recorded via different intermediate vibronic levels reveal a rich set of active cation vibrations, including fundamental modes, overtones, and combination bands. The Δν = 0 propensity rule is generally followed, confirming the structural similarity between S_1_ and D_0_ states.

Geometric parameters calculated for S_0_, S_1_, and D_0_ states reveal systematic changes upon electronic excitation (ring expansion, C-O bond shortening) and ionization (alternating ring bond length changes, further C-O contraction). These changes reflect the redistribution of electron density and the stabilization of positive charge by the electron-donating ethoxy groups.

Comparison with related molecules (phenol, anisole, phenetole, p-dimethoxybenzene, m-diethoxybenzene) reveals systematic trends in transition energies and ionization energies that correlate with the electron-donating strength and chain length of the alkoxy substituents. The para substitution pattern in PDEB leads to a more regular conformational landscape compared to the meta isomer MDEB.

This work provides comprehensive spectral and structural data for p-diethoxybenzene and deepens the understanding of structure–property relationships in diethoxybenzene isomers. The combination of high-resolution spectroscopy and quantum chemical calculations demonstrated here can be readily extended to other flexible substituted aromatic systems (such as phenetole derivatives), contributing to the fundamental understanding of molecular conformation and electronic structure.

## Figures and Tables

**Table 1:** Calculated relative zero-point-level energies (cm^−1^) of the S_0_ state rotamers of PDEB using different DFT functionals and basis sets.

Functional	Basis	cis (Up–Up)				trans (Up–Down)			
		0-0	0-Out	In–Out	Out–Out	0-0	0-Out	In–Out	Out–Out
B3PW91	6-311++G(d,p)	0	576	1172	1172	54	631	1208	1204
	aug-cc-pVTZ	0	618	1235	1236	62	666	1265	1261
B3LYP	6-311++G(d,p)	0	606	1228	1227	56	658	1256	1262
	aug-cc-pVTZ	0	633	1265	1265	60	676	1287	1295

**Table 2:** Vibrational frequencies (in cm^−1^) and assignments of observed bands in the REMPI/HB experiment of PDEB ^a^.

cis			trans			Assignment ^b^
Energy	Shift	Cal.	Energy	Shift	Cal.	
33,824	0	0	33,613	0	0	0^0^
33,937	113	108				βC_2_H_5_
			33,803	190	179	βOC_2_H_5_
34,092	268	266	33,882	269	266	νOC_2_H_5_
			34,151	538	528	6b^1^
34,385	561	558	34,204	591	594	6a^1^
34,498	674	673				17b^1^
34,634	810	809	34,426	813	812	1^1^
34,653	829					6a^1^νOC_2_H_5_
34,726	902	907	34,520	907	907	νO-CH_2_-CH_3_
34,746	922					1^1^βC_2_H_5_
			34,613	1000		1^1^βOC_2_H_5_
34,901	1077		34,692	1079		1^1^νOC_2_H_5_
34,945	1121	1124				9a^1^
35,115	1291	1275	34,900	1287	1284	7a^1^
35,193	1369		35,014	1401		1^1^6a^1^

^a^ The experimental values are shifts from 33,824 and 33,613 cm^−1^ for the cis and trans rotamers, respectively, whereas the predicted values are obtained from the B3PW91/aug-cc-pVTZ calculations, scaled by 0.97. ^b^ Internal vibrations of the substituents: ν, stretching vibrations; β, in-plane bending; γ, out-of-plane bending; δ, other bending.

**Table 3:** Vibrational frequencies (in cm^−1^) and assignments of observed bands in the MATI experiment of PDEB ^a^.

cis					trans				Assignment ^b^
Intermediate State					Intermediate State				
0	βC_2_H_5_	νOC_2_H_5_	6a	Calc.	0	βOC_2_H_5_	νOC_2_H_5_	Calc.	
109	108			104					βC_2_H_5_
					179	186		172	βOC_2_H_5_
						206			βC_2_H_5_^2^
	217								βC_2_H_5_^2^
270		274		267	266		273	267	νOC_2_H_5_
						367			βOC_2_H_5_^2^
		347							βOC_2_H_5_^2^
		386							νOC_2_H_5_βC_2_H_5_
390				391	390			390	16a^1^
							445		νOC_2_H_5_βOC_2_H_5_
		547					545		νOC_2_H_5_^2^
570			572	566					6a^1^
						574			βOC_2_H_5_16a^1^
		663							νOC_2_H_5_16a^1^
			679						6a^1^βC_2_H_5_
					776			772	10a^1^
810					813			810	1^1^
	917								βC_2_H_5_1^1^
896				897	899			898	νO-CH_2_CH_3_
						1002			βOC_2_H_5_1^1^
	1004								βC_2_H_5_νO-CH_2_CH_3_
1004				1004	1006			1004	νO-CH_2_-CH_3_
						1086			βOC_2_H_5_νO-CH_2_CH_3_
		1086					1088		νOC_2_H_5_1^1^
	1110								βC_2_H_5_νO-CH_2_-CH_3_
			1143						6a^2^
		1172					1175		νOC_2_H_5_νO-CH_2_CH_3_
1183				1171	1177			1168	9a^1^
		1278							νOC_2_H_5_νO-CH_2_-CH_3_
1291	1291								βC_2_H_5_9a^1^
						1359			βOC_2_H_5_9a^1^
			1381						6a^1^1^1^
	1400								βC_2_H_5_^2^9a^1^
		1460					1451		νOC_2_H_5_9a^1^
			1467						6a^1^νO-CH_2_CH_3_
			1574						6a^1^νO-CH_2_-CH_3_
1647				1652	1642			1648	8a^1^
					1712				1^1^νO-CH_2_CH_3_
			1754						6a^1^9a^1^
	1757								βC_2_H_5_8a^1^

^a^ The experimental values are shifts from 59,629 and 59,432 cm^−1^ for cis and trans rotamers, respectively, whereas the predicted values are obtained from UB3PW91/aug-cc-pVTZ calculations, scaled by 0.98. ^b^ Internal vibrations of the substituents: ν, stretching vibrations; β, in-plane bending; γ, out-of-plane bending; δ, other bending.

**Table 4:** Geometric parameters of the cis rotamer of PDEB in the S_0_, S_1_, and D_0_ states calculated at B3PW91/aug-cc-pVTZ, TD-B3PW91/aug-cc-pVTZ, and UB3PW91/aug-cc-pVTZ levels, respectively.

	S_0_	S_1_	D_0_	Δ(S_1_ − S_0_)	Δ(D_0_ − S_1_)
Bond length (Å)					
C1-C2	1.397	1.414	1.425	0.017	0.011
C2-C3	1.377	1.413	1.355	0.036	−0.058
C3-C4	1.397	1.414	1.425	0.017	0.011
C4-C5	1.387	1.412	1.420	0.025	0.008
C5-C6	1.397	1.416	1.365	0.019	−0.051
C6-C1	1.387	1.412	1.420	0.025	0.008
C1-O11	1.362	1.342	1.306	−0.02	−0.036
O11-C13	1.417	1.428	1.457	0.011	0.029
C13-C14	1.510	1.508	1.503	−0.002	−0.005
C4-O12	1.362	1.342	1.306	−0.02	−0.036
O12-C20	1.417	1.428	1.457	0.011	0.029
C20-C21	1.510	1.508	1.503	−0.002	−0.005
Bond angle (°)					
C1C2C3	119.81	115.94	118.68	−3.88	2.74
C2C3C4	120.13	122.34	120.56	2.20	−1.78
C3C4C5	119.18	120.99	120.30	1.81	−0.69
C4C5C6	121.73	117.46	119.81	−4.28	2.36
C5C6C1	118.58	120.64	119.86	2.06	−0.78
C6C1C2	120.56	122.46	120.79	1.90	−1.67
C6C1O11	124.48	123.28	122.70	−1.20	−0.58
C1O11C13	118.42	120.28	122.65	1.86	2.37
O11C13C14	107.96	107.94	107.49	−0.02	−0.45
C4C3O12	115.89	114.53	113.53	−1.36	−1.00
C3O12C20	118.60	119.53	120.50	0.93	0.97
O12C20C21	107.96	107.97	108.01	0.02	0.04
Other parameters					
Point group	C2v	Cs	C2v		

**Table 5:** Geometric parameters of the trans rotamer of PDEB in the S_0_, S_1_, and D_0_ states calculated at B3PW91/aug-cc-pVTZ, TD-B3PW91/aug-cc-pVTZ, and UB3PW91/aug-cc-pVTZ levels, respectively.

	S_0_	S_1_	D_0_	Δ(S_1_ − S_0_)	Δ(D_0_ − S_1_)
Bond length (Å)					
C1-C2	1.394	1.408	1.419	0.014	0.011
C2-C3	1.387	1.414	1.361	0.027	−0.053
C3-C4	1.390	1.417	1.425	0.027	0.008
C4-C5	1.394	1.408	1.419	0.014	0.011
C5-C6	1.387	1.414	1.361	0.027	−0.053
C6-C1	1.390	1.417	1.425	0.027	0.008
C1-O11	1.362	1.342	1.307	−0.02	−0.035
O11-C13	1.417	1.427	1.457	0.01	0.03
C13-C14	1.510	1.508	1.503	−0.002	−0.005
C4-O12	1.362	1.342	1.307	−0.02	−0.035
O12-C20	1.417	1.427	1.457	0.01	0.03
C20-C21	1.510	1.508	1.503	−0.002	−0.005
Bond angle (°)					
∠C1C2C3	120.1	118.1	119.3	−2	1.2
∠C2C3C4	121	119.1	120.8	−1.9	1.7
∠C3C4C5	118.9	122.8	119.9	3.9	−2.9
∠C4C5C6	120.1	118.1	119.3	−2	1.2
∠C5C6C1	121	119.1	120.8	−1.9	1.7
∠C6C1C2	118.9	122.8	119.9	3.9	−2.9
∠C2C1O11	124.7	123.2	124.9	−1.5	1.7
∠C1O11C13	118.3	120.4	121.7	2.1	1.3
∠O11C13C14	108	107.9	107.8	−0.1	−0.1
∠C5C4O12	124.7	123.2	124.9	−1.5	1.7
∠C4O12C20	118.3	120.4	121.7	2.1	1.3
∠O12C20C21	108	107.9	107.8	−0.1	−0.1
Other parameters					
Point group	C2h	C1	C2h		

**Table 6:** Measured transition energies (cm^−1^) and ionization energies of several benzene derivatives.

Molecule	S_1_←S_0_	ΔE_1_ ^a^	IE	ΔIE ^b^	References
Benzene	38,086	0	74,557	0	[51,52]
Anisole	36,383	−1703	66,399	−8158	[53]
Phenetole	36,370	−1716	65,890	−8667	[40]
p-Dimethoxybenzene (cis)	33,852	−4234	60,772	−13,785	[42]
p-Dimethoxybenzene (trans)	33,631	−4455	60,563	−13,994	[42]
p-Diethoxybenzene (cis)	33,824	−4262	59,629	−14,928	This work
p-Diethoxybenzene (trans)	33,613	−4473	59,432	−15,125	This work
m-Diethoxybenzene (down-up)	36,091	−1995	62,419	−12,138	[3]
m-Diethoxybenzene (down-down)	36,165	−1921	63,378	−11,179	[3]

^a^ ΔE_1_ = shift in S_1_←S_0_ transition energy relative to benzene. ^b^ ΔIE = shift in ionization energy relative to benzene.

## Data Availability

The data that support the findings of this study are available from the corresponding author, Changyong Li, upon reasonable request.

## Supplementary Materials

The following supporting information can be downloaded at: https://www.mdpi.com/article/10.3390/ijms27083362/s1.

## References

[1] Cinacchi G., Prampolini G. (2003). DFT Study of the Torsional Potential in Ethylbenzene and Ethoxybenzene: The Smallest Prototypes of Alkyl− and Alkoxy−Aryl Mesogens. J. Phys. Chem. A.

[2] Li S., Zhao Y., Hu F., Jiao Y., Zhao J., Li C. (2023). The stable conformations and vibronic and cation spectroscopy of 2-ethoxybenzonitrile. J. Mol. Struct..

[3] Qin X., Duan C., Zhao Y., Li C., Jia S. (2025). Study of Vibronic and Cationic Features of m-Diethoxybenzene via REMPI, Hole-Burning, and MATI Spectroscopy. Int. J. Mol. Sci..

[4] Huang J., Li C., Tzeng W.B. (2005). Mass analyzed threshold ionization spectroscopy of p-methylanisole cation and the substitution effect. Chem. Phys. Lett..

[5] Huang J., Lin J.L., Tzeng W.B. (2006). Mass analyzed threshold ionization spectroscopy of the 35Cl and 37Cl isotopomers of p-chlorophenol and isotope effect. Chem. Phys. Lett..

[6] Huang J., Lin J.L., Tzeng W.B. (2007). Rotamers of m-cresol cation studied by mass-analyzed threshold ionization spectroscopy. Spectrochim. Acta A Mol. Biomol. Spectrosc..

[7] Huang J., Huang K., Liu S., Luo Q., Tzeng W. (2007). Molecular structures and vibrations of cis and trans m-cresol in the electronically excited S_1_ and cationic D_0_ states. J. Photochem. Photobiol. A Chem..

[8] Andrejeva A., Tuttle W.D., Harris J.P., Wright T.G. (2015). Resonance-enhanced multiphoton ionization (REMPI) spectroscopy of bromobenzene and its perdeuterated isotopologue: Assignment of the vibrations of the S, S^1^, and D^+^ states of bromobenzene and the S and D^+^ states of iodobenzene. J. Chem. Phys..

[9] Tuttle W.D., Gardner A.M., Wright T.G. (2017). Resonance-enhanced multiphoton ionization (REMPI) spectroscopy of p–chlorofluorobenzene. Chem. Phys. Lett..

[10] He Y., Wu C., Kong W. (2004). Observation of rotamers of m-aminobenzoic acid: Zero kinetic energy photoelectron and hole-burning resonantly enhanced multiphoton ionization spectroscopy. J. Chem. Phys..

[11] Harthcock C., Zhang J., Kong W., Mitsui M., Ohshima Y. (2017). Electronic spectra and excited-state dynamics of acridine and its hydrated clusters. J. Chem. Phys..

[12] Choi C.M., Choi D.H., Heo J., Kim N.J., Kim S.K. (2012). Ultraviolet-ultraviolet hole burning spectroscopy in a quadrupole ion trap: Dibenzo18crown-6 complexes with alkali metal cations. Angew. Chem. Int. Ed. Engl..

[13] Kang H., Féraud G., Dedonder-Lardeux C., Jouvet C. (2014). New Method for Double-Resonance Spectroscopy in a Cold Quadrupole Ion Trap and Its Application to UV-UV Hole-Burning Spectroscopy of Protonated Adenine Dimer. J. Phys. Chem. Lett..

[14] Yoo I.T., Jeong J., Eun H.J., Yun J., Heo J., Kim N.J. (2024). Conformation-Selective Ultraviolet-Ultraviolet Hole Burning Spectra of Ubiquitin Ions in a Cryogenic Ion Trap. J. Phys. Chem. Lett..

[15] Kalal B., Maity S. (2026). Non-Traditional Excited-State Deactivation in N-Containing Chromophores: A Combined Spectroscopic and Computational Study. J. Phys. Chem. Lett..

[16] Singh S.K., Mishra K.K., Sharma N., Das A. (2016). Direct Spectroscopic Evidence for an n→π* Interaction. Angew. Chem. Int. Ed. Engl..

[17] Zhu L., Johnson P. (1991). Mass analyzed threshold ionization spectroscopy. J. Chem. Phys..

[18] Park S.M., Kim H., Kwon C.H. (2025). Unraveling the Conformational and Electronic Landscape of 3-Pyridinecarboxaldehyde: A Spectroscopic Journey through Conformer-Specific Ionization. ACS Phys. Chem. Au.

[19] Kim H., Park S.M., Kwon C.H. (2025). Uncovering the role of fluorine positioning on the cationic properties of 2,4-difluoropyridine. Phys. Chem. Chem. Phys..

[20] Kim H., Park S.M., Kwon C.H. (2025). Resolving ionization-induced distortions in 2,5-difluoropyridine: Influences of meta-fluorination on electronic and vibrational structures. Phys. Chem. Chem. Phys..

[21] Kim H., Park S.M., Kwon C.H. (2025). Fluorine position-dependent ionization dynamics and structural distortion in 2,3-difluoropyridine: A VUV-MATI and theoretical study. Phys. Chem. Chem. Phys..

[22] Kim H., Park S.M., Kwon C.H. (2025). Deciphering the electronic structure and conformational stability of 2-pyridinecarboxaldehyde. Phys. Chem. Chem. Phys..

[23] Nyambo S., Zhang Y., Yang D.-S. (2025). Reactions of La and Ce with Propylamine: Formation and Threshold Ionization of Lanthanide Imido Radicals. J. Phys. Chem. A.

[24] Ketkov S.Y., Tzeng S.-Y., Rychagova E.A., Lukoyanov A.N., Tzeng W.-B. (2024). Effect of a single methyl substituent on the electronic structure of cobaltocene studied by computationally assisted MATI spectroscopy. Phys. Chem. Chem. Phys..

[25] Ketkov S.Y., Tzeng S.-Y., Rychagova E.A., Lukoyanov A.N., Markin G.V., Tzeng W.-B. (2025). Intriguing methylation effects in cobaltocene revealed by high-resolution MATI spectroscopy and ab initio calculations of (η^5^-C_5_H_4_Me)_2_Co. J. Chem. Phys..

[26] Huang J., Huang K., Liu S., Luo Q., Tzeng W. (2008). Vibrational spectra and theoretical calculations of p-chlorophenol in the electronically excited S_1_ and ionic ground D_0_ states. J. Photochem. Photobiol. A Chem..

[27] Park S.M., Lee Y.R., Kwon C.H. (2022). Conformational Structures of Neutral and Cationic Pivaldehyde Revealed by IR-Resonant VUV-MATI Mass Spectroscopy. Int. J. Mol. Sci..

[28] Müller-Dethlefs K., Sander M., Schlag E.W. (1984). Two-colour photoionization resonance spectroscopy of NO: Complete separation of rotational levels of NO^+^ at the ionization threshold. Chem. Phys. Lett..

[29] Zhang J., Harthcock C., Kong W. (2012). Resonantly enhanced multiphoton ionization and zero kinetic energy photoelectron spectroscopy of chrysene: A comparison with tetracene. J. Phys. Chem. A.

[30] Harthcock C., Zhang J., Kong W. (2014). Zero kinetic energy photoelectron spectroscopy of triphenylene. J. Chem. Phys..

[31] Harthcock C., Zhang J., Kong W. (2015). Zero Kinetic Energy Photoelectron Spectroscopy of Benzohquinoline. J. Phys. Chem. A.

[32] Kreis C., Schmitz J.R., Merkt F. (2024). Characterisation of the ground X^+ 2^Π_Ω_ and first excited A^+ 2^Σ^+^ electronic states of MgO^+^ by high-resolution photoelectron spectroscopy. Phys. Chem. Chem. Phys..

[33] Herburger H., Wirth V., Hollenstein U., Merkt F. (2023). Pulsed-ramped-field-ionisation zero-kinetic-energy photoelectron spectroscopy of the metastable rare-gas atoms Ar, Kr and Xe. Phys. Chem. Chem. Phys..

[34] Zhao Y., Jin Y., Hao J., Yang Y., Wang L., Li C., Jia S. (2019). Rotamers of p-isopropylphenol studied by hole-burning resonantly enhanced multiphoton ionization and mass analyzed threshold ionization spectroscopy. Spectrochim. Acta A Mol. Biomol. Spectrosc..

[35] Ullrich S., Geppert W.D., Dessent C.E.H., Müller-Dethlefs K. (2000). Observation of Rotational Isomers I: A ZEKE and Hole-Burning Spectroscopy Study of 3-Methoxyphenol. J. Phys. Chem. A.

[36] Xu Y., Tzeng S.Y., Shivatare V., Takahashi K., Zhang B., Tzeng W.B. (2015). Identification of four rotamers of m-methoxystyrene by resonant two-photon ionization and mass analyzed threshold ionization spectroscopy. J. Chem. Phys..

[37] Fan J., Finazzi L., Jan Buma W. (2022). Elucidating the photoprotective properties of natural UV screening agents: ZEKE-PFI spectroscopy of methyl sinapate. Phys. Chem. Chem. Phys..

[38] Bernstein E.R., Im H.S., Young M.A., Secor H.V., Bassfield R.L., Seeman J.I. (1991). Supersonic jet studies of ethoxybenzenes: Geometry of their minimum energy conformations. J. Org. Chem..

[39] Ferres L., Stahl W., Nguyen H.V.L. (2016). The molecular structure of phenetole studied by microwave spectroscopy and quantum chemical calculations. Mol. Phys..

[40] Helle N., Hintelmann I., Grotemeyer J. (2019). Detailed analysis of the vibronic structure of phenetole in its first excited state and ionic ground state. Eur. J. Mass Spectrom..

[41] Zheng Q., Fang I.T., Zhang B., Bih Tzeng W. (2009). Mass-analyzed Threshold Ionization Spectroscopy of Rotamers of p -ethoxyphenol Cations and Configuration Effect. Chin. J. Chem. Phys..

[42] Lin J.L., Huang L.C.L., Tzeng W.B. (2001). Mass-Analyzed Threshold Ionization Spectroscopy of the Selected Rotamers of Hydroquinone and p -Dimethoxybenzene Cations. J. Phys. Chem. A.

[43] Tzeng W.B., Narayanan K., Hsieh C.Y., Tung C.C. (1998). Structures and vibrations of p-dimethoxybenzene conformers in the S_0_ and S_1_ states studied by ab initio calculations and resonant two-photon ionization spectroscopy. J. Mol. Struct..

[44] Yamamoto S., Okuyama K., Mikami N., Ito M. (1986). Selective complexation of rotational isomers of p-dimethoxybenzene as studied by electronic spectra in a supersonic jet. Chem. Phys. Lett..

[45] Varsanyi G. (1974). Assignments of Vibrational Spectra of Seven Hundred Benzene Derivatives.

[46] Varsanyi G., Szoke S. (1969). Vibrational Spectra of Benzene Derivatives.

[47] Bright Wilson E. (1934). The Normal Modes and Frequencies of Vibration of the Regular Plane Hexagon Model of the Benzene Molecule. Phys. Rev..

[48] Li C., Su H., Tzeng W.B. (2005). Rotamers of p-methoxyphenol cation studied by mass analyzed threshold ionization spectroscopy. Chem. Phys. Lett..

[49] Li S., Zhao Y., Jiao Y., Zhao J., Li C., Jia S. (2023). Vibronic and Cationic Features of 2-Fluorobenzonitrile and 3-Fluorobenzonitrile Studied by REMPI and MATI Spectroscopy and Franck-Condon Simulations. Molecules.

[50] Li N., Li S., Wang L., Wang H., Zhao J., Li C. (2022). Vibrational spectra of 2-cyanophenol cation studied by the mass analyzed threshold ionization technique. Chem. Phys. Lett..

[51] Neuhauser R.G., Siglow K., Neusser H.J. (1997). High n Rydberg spectroscopy of benzene: Dynamics, ionization energy and rotational constants of the cation. J. Chem. Phys..

[52] Chewter L.A., Sander M., Müller-Dethlefs K., Schlag E.W. (1987). High resolution zero kinetic energy photoelectron spectroscopy of benzene and determination of the ionization potential. J. Chem. Phys..

[53] Pradhan M., Li C., Lin J.L., Tzeng W.B. (2005). Mass analyzed threshold ionization spectroscopy of anisole cation and the OCH_3_ substitution effect. Chem. Phys. Lett..

[54] Frisch M.J., Trucks G.W., Schlegel H.B., Scuseria G.E., Robb M.A., Cheeseman J.R., Scalmani G., Barone V., Mennucci B., Petersson G.A. (2016). Gaussian 16 Revision C.01.

[55] Becke A.D. (1993). Density-functional thermochemistry. III. The role of exact exchange. J. Chem. Phys..

[56] Perdew J.P., Wang Y. (1992). Accurate and simple analytic representation of the electron-gas correlation energy. Phys. Rev. B Condens. Matter.

[57] Dunning T.H. (1989). Gaussian basis sets for use in correlated molecular calculations. I. The atoms boron through neon and hydrogen. J. Chem. Phys..

[58] Casida M.E., Chong D.P. (1995). Time-Dependent Density Functional Response Theory for Molecules. Recent Advances in Density Functional Methods.

[59] Runge E., Gross E.K.U. (1984). Density-Functional Theory for Time-Dependent Systems. Phys. Rev. Lett..

[60] Stephens P.J., Devlin F.J., Chabalowski C.F., Frisch M.J. (1994). Ab Initio Calculation of Vibrational Absorption and Circular Dichroism Spectra Using Density Functional Force Fields. J. Phys. Chem..

