# Supplementary material for: Study of Vibronic and Cationic Features of p-Diethoxybenzene via REMPI, Hole-Burning, and MATI Spectroscopy
Source: Int J Mol Sci. 2026 Apr 9;27(8):3362. doi: 10.3390/ijms27083362 (PMC13116328; doi:10.3390/ijms27083362)
Supplement: Supplementary file 1 [file ijms-27-03362-s001.zip › ijms-4225974-supplementary.pdf]

Supporting Information

Study of Vibronic and Cationic Features of p-Diethoxybenzene via REMPI, Hole-Burning, and MATI Spectroscopy

Xiateng Qin [1], Yan Zhao [2], Zhonghua Ji [1,3], Changyong Li [1,3,*] and Suotang Jia [1,3]

1 State Key Laboratory of Quantum Optics Technologies and Devices, Institute of Laser Spectroscopy, Shanxi University, Taiyuan 030006, China; 18734558738@163.com (X.Q.); jzh@sxu.edu.cn (Z.J.); tjia@sxu.edu.cn (S.J.)
2 Department of Physics and Electronics Engineering, Jinzhong University, Jinzhong 030619, China; zhaoy@jzxy.edu.cn
3 Collaborative Innovation Center of Extreme Optics, Shanxi University, Taiyuan 030006, China
* Correspondence: lichyong@sxu.edu.cn

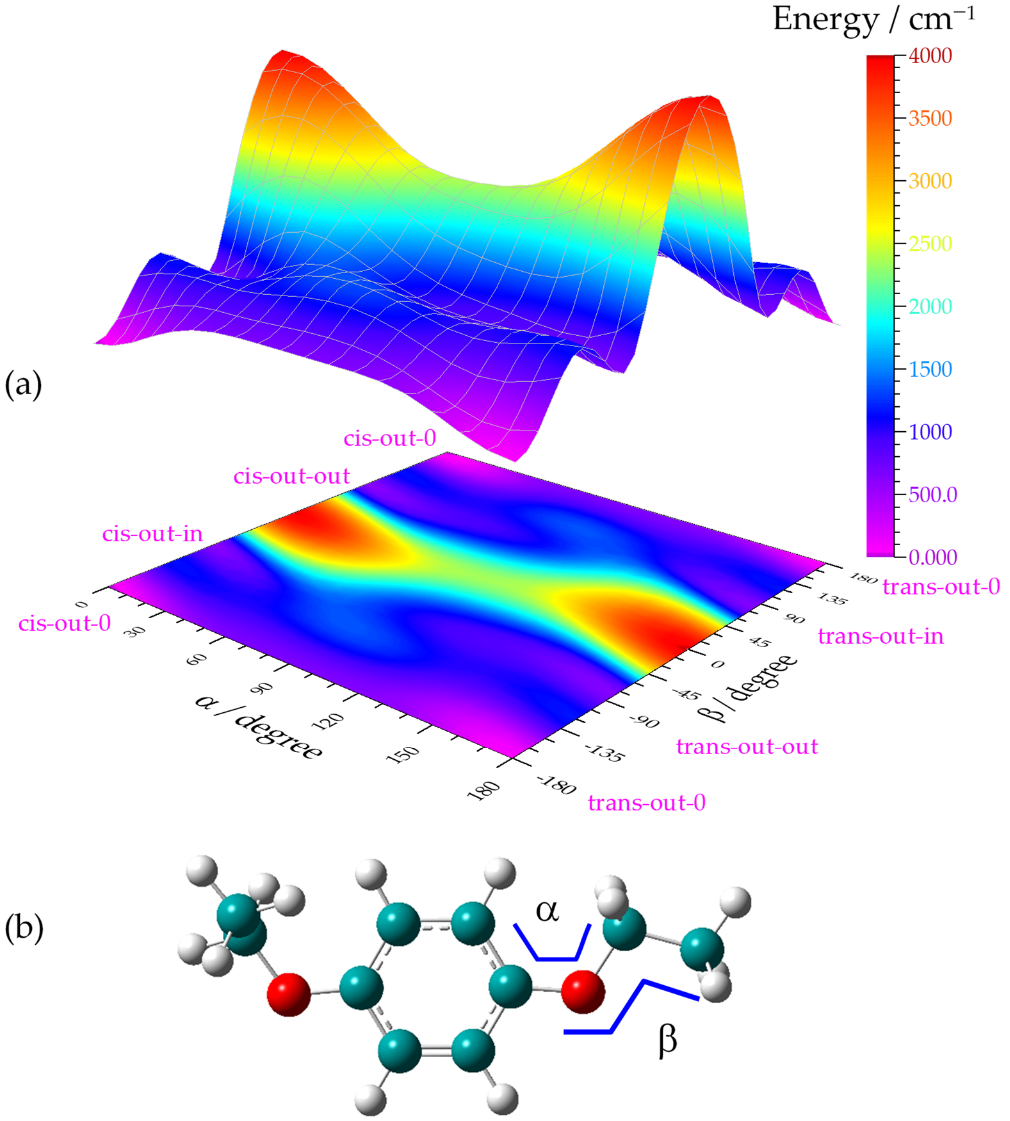

Figure S1. The potential energy surface (PES) calculated at the B3LYP/6-311++G(d,p) level for the S_0 state of para-diethoxybenzene (a), and the two dihedral angles α and β used for the PES scan (b). Eight local minima are identified in the figure, corresponding to the six rotamers of para-diethoxybenzene: cis-out-0 (or cis-0-out), cis-out–out, cis-out–in (or cis-in–out), trans-out-0 (or trans-0-out), trans-out–out, and trans-out–in (or trans-in–out), respectively.
